# The value of color doppler ultrasonography combined with computed tomography angiography and magnetic resonance angiography in the preoperative quantification and classification of carotid body tumors: a retrospective analysis

**DOI:** 10.1186/s12880-023-01189-x

**Published:** 2024-01-03

**Authors:** Li Zhiqiang, Wang Yihua, Fu Ying, Zhu Shiwei, Zeng Xiangzhu, Cui Ligang

**Affiliations:** 1 Department of Ultrasound Medicine, Peking University Third Hospital, 49 North Garden Rd., Haidian District, Beijing, 100191 P.R. China; 2 Department of Ultrasound, North China University of Science and Technology Affiliated Hospital, 73 South Jianshe Rd, Lubei District, Tangshan, 063000 P.R. China; 3 Department of Radiology, Peking University Third Hospital, 49 North Garden Rd., Haidian District, Beijing, 100191 P.R. China

**Keywords:** Carotid body tumor, Color doppler ultrasound, Computed tomography angiography, Magnetic resonance angiography

## Abstract

**Background:**

Computed tomography angiography (CTA) and magnetic resonance angiography (MRA) provide accurate vascular imaging information, but their use may be contraindicated. Color Doppler ultrasonography (CDU) provides simple, safe, noninvasive, and reproducible imaging. We therefore investigated the role of preoperative CDU combined with CTA and MRA in the quantification, typing, and diagnosis of carotid body tumors (CBTs).

**Methods:**

We retrospectively analyzed patients with CBTs categorized into group A (type I [n = 1] and type II [n = 10]) or group B (type III [n = 56]) per the intraoperative Shamblin classification. CDU, CTA, and MRA characteristics of CBTs were observed, surgical results were correlated, and the diagnostic threshold of the CBT classification was calculated.

**Results:**

CBTs were usually located at the common carotid artery bifurcation, encircling the carotid artery. An increased angle was found between the internal and external carotid arteries. On CDU, CBTs primarily presented as homogeneous hypoechoic masses with clear boundaries, rich flow signals, and a high-speed, low-resistance artery-like flow spectrum. CTA showed uniform or heterogeneous marked enhancement. MRA showed mixed T1 and slightly longer T2 signals and uniform or uneven obvious enhancement. With increases in the lesion size, amount of blood transfused, and operation time, the intraoperative classification level and possibility of skull-base invasion increased. When the maximum diameter of the lesion, the volume of the tumor, the distance between the upper margin of the tumor to the mastoid and the mandibular angle were 3.10 cm, 10.15 cm^3^, − 3.26 cm, and 0.57 cm, respectively, the largest Youden index was the best diagnostic boundary value for Shamblin type III tumors.

**Conclusions:**

CDU combined with CTA and MRA can accurately evaluate the size and classification of CBTs.

## Background

The carotid body, the largest accessory ganglion in the human body, is a peripheral respiratory receptor approximately 3 to 3.5 mm in diameter. Connective tissue attaches the carotid body at the medial or posterior side of the bifurcation of the common carotid artery and it is mainly supplied by the external carotid artery. Carotid body tumors (CBTs) are rare, slow-growing, highly vascular, nonchromaffin neurovascular tumors [1]: they account for more than half of head and neck paragangliomas and have an incidence of 1 in 30,000.

Patients with CBTs are usually asymptomatic and the tumors are usually benign; however, some tumors have a malignant tendency. With tumor enlargement, patients eventually develop clinical symptoms. Early surgical treatment is recommended to reduce invasion of the peripheral blood vessels and nerves. As the tumor is highly vascularized, closely related to the carotid bifurcation, and strongly adherent to the carotid artery wall, postoperative complications such as bleeding, cranial nerve injury, and stroke may occur [2]. Reliable preoperative imaging evaluation, classification, and differential diagnosis are crucial. Shamblin et al. classified CBTs based on tu-mor size and carotid artery involvement [3]. Type I tumors have a small volume and only a small part of the tumor is in contact with the carotid artery. Type II tumors are large and much of the tumor is in contact with the carotid artery. Type III tumors are large and completely surround the carotid artery.

Computed tomography angiography (CTA) and magnetic resonance angiography (MRA) can provide accurate vascular imaging information; however, their use may be contraindicated. Color Doppler ultrasonography (CDU) provides simple, safe, noninvasive, and reproducible imaging. Recently, ultrasound imaging technology has developed rapidly and is now widely used in the diagnosis and evaluation of CBTs. In this study, we reviewed the CDU, CTA, and MRA characteristics of different types of CBT and analyzed their correlation with surgical results to explore the role of CDU combined with CTA and MRA in the preoperative quantification, typing, and diagnosis of CBT.

## Methods

### Patients

This retrospective study evaluated 66 patients (67 lesions) with a pathological diagnosis of CBT who underwent surgical resection at the Peking University Third Hospital between May 2017 and December 2022. Depending on the Shamblin typing of the tumors, patients were assigned to groups A (Shamblin types I and II) or B (Shamblin type III).

All patients underwent preoperative CDU, CTA, and MRA. This study was approved by the Ethics Committee of Peking University Third Hospital (Approval number: IRB00006761-M2023379) and did not require images, records, or informed consent from patients.

### Procedure

General data were gathered regarding patient age, sex, and clinical manifestations. Surgical data collected included the total hospitalization time, surgical blood loss, blood transfused during surgery, and duration of surgery; CBT data included intraoperative lesion location, color, quality, texture, capsule, relationship with the internal and external carotid arteries of the bifurcated neck, blood supply, and skull-base invasion occurrence. Collected pathological data included the findings of gross pathology, immunohistochemistry, and molecular pathology.

Intraoperatively, each CBT was categorized using Shamblin classification into one of three types based on the connection between the tumor and the carotid artery [3]. Type I tumors have a small volume and only a small part of the tumor is in contact with the carotid artery; they can be surgically resected. Type II tumors are large and much of the tumor is in contact with the carotid artery; the tumor can be removed, although temporary intracavitary carotid bypass may be required. Type III tumors are large and completely surround the carotid artery; frequently, carotid artery resection and vascular transplantation are required.

Images from the final preoperative examination were analyzed by both a senior (15years) and junior (10years) diagnostic physician. The physicians were blinded to the patient’s clinical information, imaging findings, and pathology results. They reviewed the images and videos. The final diagnosis was determined through consultation between the two physicians. In cases of disagreement, a senior sonologist with 20 years reviewed the case and made the final decision.

Imaging parameters, including the lesion location, number, density, boundary, morphology, enhancement pattern, degree of contact with the carotid artery, carotid artery bifurcation angle, changes in the carotid artery morphology, and blood flow to the lesion, were classified. CDU was performed with the SAMSUNG RS80A (Samsung Medison, Gyeonggi-do, South Korea), LOGIQ E9(GE Healthcare, Wauwatosa, WI, USA) and Acuson Sequoia 512(Siemens Healthcare, Erlangen, Germany) ultrasound systems. Adler classification was used to determine blood flow to the lesion: grade 0 indicates no blood flow signal; grade 1, low blood flow with visible dotted blood flow at one or two points; grade 2, moderate blood flow, with several small vessels or one vessel exceeding the radius of the lesion; and grade 3, rich blood flow, with more than four vessels or an interwoven network of blood vessels visible. CTA imaging was performed using a multi-slice spiral CT scanner (Discover uCT 960+, United Imaging Healthcare, Shanghai, China). All cases were evaluated preoperatively using CTA (slice thickness, 0.625 mm). The upper and lower diameters of the tumor (2a), left and right diameters (2b), and front and rear diameters (2c) were measured, and the ellipsoid volume was used to calculate the tumor volume using the formula volume = 4πabc/3. Two physicians digitally measured and calculated the volume. The upper margin of the tumor, skull base, mastoid, and mandibular angle were identified by axial projection, and the distances from the upper margin of the tumor to the mastoid and mandibular angle were calculated. MRA imaging was performed using a 3.0T magnetic resonance equipment (uMR780, United Imaging Healthcare, Shanghai, China). MRA was performed in all cases using a 3.0T magnet. Axial and coronal spin-echo T1-weighted; axial and sagittal fast spin-echo T2-weighted; coronal and sagittal short tau inversion recovery; and contrast axial, coronal, and sagittal T1-weighted sequenced images were captured. A 4 mm-thick sequence was obtained in the absence of crossover clearance. MR angiography was performed in all cases with a 0.1 m mol/kg gadolinium-diethyltriaminopentanoacetate injection. The matrix was 256 × 256 and the field of view was 220 mm. A two- or three-dimensional time of flight sequence was used (repetition time, 25–35 ms; echo time, 4–8 ms) with a slice thickness of 1 mm.

### Statistical analysis

A Microsoft Excel 2017 database was established comprising all the data collected; statistical analyses were conducted using IBM SPSS Statistics for Windows version 22.0 (IBM Corp.; Armonk NY, USA). Normally distributed measurement data were expressed as $(\bar{x}\pm \delta )$ and biased distribution data as median (interquartile range) using the Mann–Whitney U test. Spearman’s correlation and Kendall’s tau-b (K) correlation analyses (r ≥ 0.8, high correlation; 0.8 > r ≥ 0.5, moderate correlation; 0.5 > r ≥ 0.3, low correlation) were used. Count data adoption rates were expressed, and between-group comparisons were performed using the χ^*2*^ and Fisher Exact tests. Consistency of diagnosis was evaluated using the Kappa test (Kappa value > 0.75, good consistency; Kappa value = 0.40 to 0.75, moderate consistency; Kappa value < 0.40, poor consistency). The diagnostic efficacy of the regression model was analyzed using receiver operating characteristic (ROC) curves. An area under the curve of < 0.5 indicated an unreliable diagnosis; that of 0.5 to 0.7 indicated low diagnostic accuracy; that of 0.7 to 0.9 indicated moderate diagnostic accuracy; and that of > 0.9 indicated high diagnostic accuracy. Differences in the area under the curve were analyzed using the DeLong test. Statistical significance was defined as *P* < 0.05.

## Results

### Clinical data analysis

Fifty-seven (86.36%) patients had unilateral CBT onset and nine (13.64%) had bilateral onset. One patient underwent bilateral staging surgery and eight underwent unilateral surgery.

A comparison of the general data between the two groups (Table 1) showed no significant difference in sex, age, clinical manifestations, or total hospital stay between the two groups (*P* > 0.05). The number of foci invading the skull base was greater in group B than in group A, with greater surgical blood loss, larger blood transfusion volumes, and longer operation time (*P* < 0.05).

**Table 1:** Analysis of the clinical data for carotid body tumors in groups A and B

		Group A (n = 11)	Group B (n = 56)	*χ*^2^/*Z*	*P*
Sex	Male	5 (45.5)	28 (50.0)	0.076	0.783
	Female	6 (54.5)	28 (50.0)		
Clinical manifestation	Yes	6 (54.5)	32 (57.1)	0.025	0.874
	No	5 (45.5)	24 (42.9)		
Dizzy	Yes	1 (9.1)	3 (5.4)		0.521
	No	10 (90.0)	53 (94.6)		
Headache	Yes	2 (18.2)	1 (1.8)		0.068
	No	9 (81.8)	55 (98.2)		
Pharyngeal discomfort	Yes	1 (9.1)	12 (21.4)		0.317
	No	10 (90.9)	44 (78.6)		
Alalia	Yes	0 (0.0)	1 (1.8)		0.836
	No	11 (100.0)	55 (98.2)		
Abnormal swallowing	Yes	1 (9.1)	2 (3.6)		0.421
	No	10 (90.9)	54 (96.4)		
Swelling of the neck	Yes	3 (27.3)	5 (8.9)		0.117
	No	8 (72.7)	51 (91.1)		
Throat pain	Yes	0 (0.0)	2 (3.6)		0.697
	No	11 (100.0)	54 (96.4)		
Hoarseness	Yes	1 (9.1)	1 (1.8)		0.303
	No	10 (90.9)	55 (98.2)		
Drinking water choking cough	Yes	1 (9.1)	8 (14.3)		0.543
	No	10 (90.9)	48 (85.7)		
Cough	Yes	2 (18.2)	9 (16.1)		0.579
	No	9 (81.8)	47 (83.9)		
Expiratory dyspnea	Yes	0 (0.0)	2 (3.6)		0.697
	No	11 (100.0)	54 (96.5)		
Facial numbness	Yes	0 (0.0)	1 (1.8)		0.836
	No	11 (100.0)	55 (98.2)		
Skull-base invasion	Yes	1 (9.1)	37 (66.1)		0.001
	No	10 (90.9)	19 (33.9)		
Age (years)		46.00 (37.00, 56.00)	41.50 (31.00, 51.75)	1.000	0.318
Total length of hospital stay (days)		11.00 (7.00, 14.00)	10.00 (7.00, 17.00)	0.154	0.878
Surgical blood loss (mL)		5.00 (5.00, 5.00)	50.00 (12.50, 150.00)	4.534	<0.001
Surgical blood transfusion volume (mL)		0.00 (0.00, 0.00)	5.00 (0.00, 120.00)	2.911	0.004
Duration of surgery (hours)		136.00 (110.00, 160.00)	239.50 (164.25, 452.50)	3.081	0.002

### CDU combined with CTA and MRA analysis

The Kappa consistency test of the CBT imaging classification yielded a value of 0.884 (*P* = 0.000, < 0.05), indicating good agreement of the imaging classification between the senior and junior physicians (Table 2).

**Table 2:** Agreement analysis of carotid body tumor imaging Shamblin classification between two physicians

		Junior physician		
		Type I	Type II	Type III
Senior physician	Type I	1	0	0
	Type II	0	13	0
	Type III	0	3	49

CBTs were usually located at the bifurcation of the common carotid artery and consisted mostly of a round or ovoid mass. Imaging showed that most (87.5%) lesions in group B completely surrounded the bifurcation, internal carotid artery, and origin of the external carotid artery. Ten lesions in group A partially surrounded the bifurcation (Shamblin type II) and one lesion did not (Shamblin type I). In both groups, the angles between the internal and external carotid arteries increased toward the affected side, and the arteries were either displaced or slightly displaced.

**Fig. 1:**
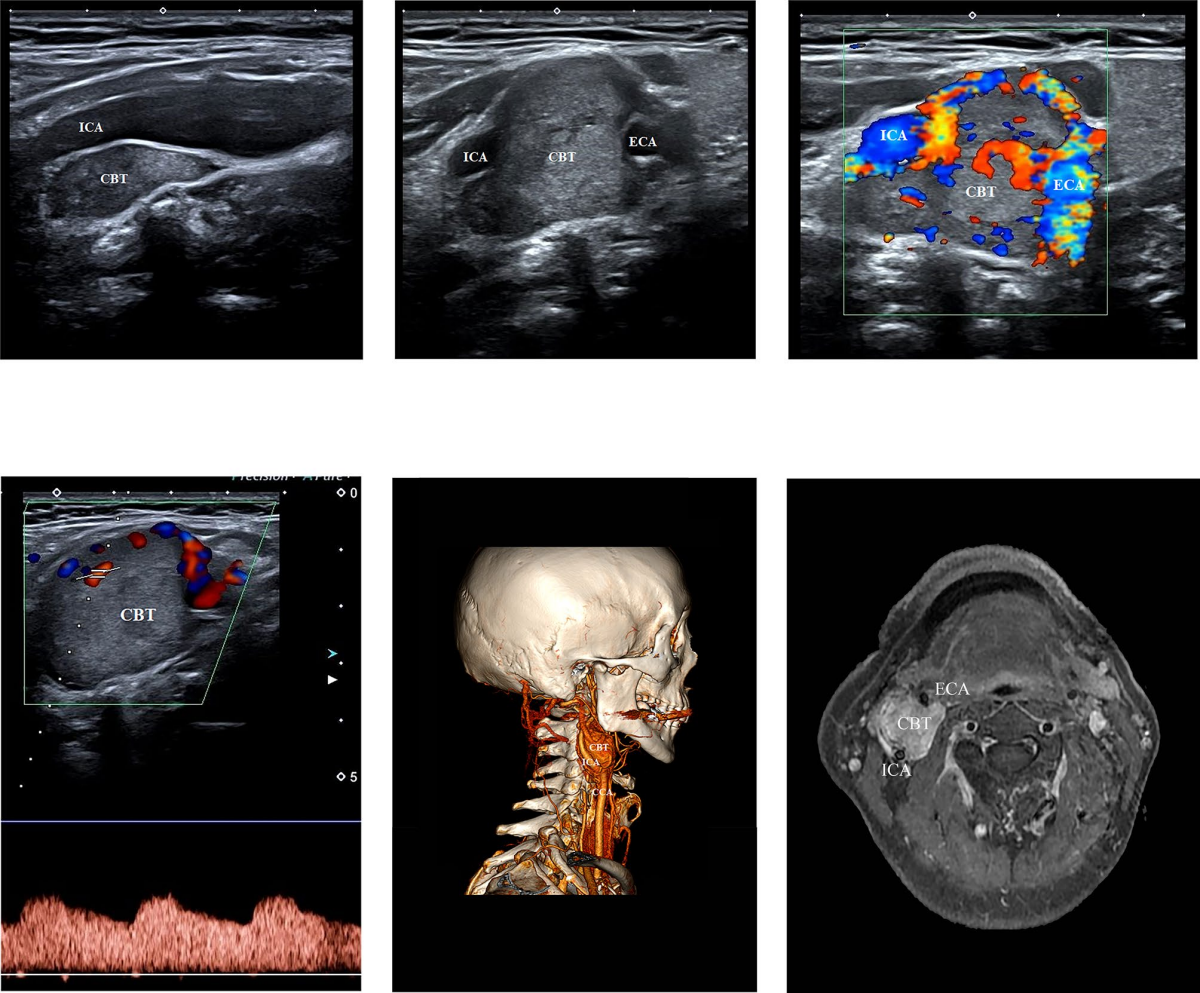
Image data of CBTs were collected on gray-scale, CDU, CTA, and MRA. (**a**) Gray scale ultrasound image of a Shamblin III CBT on sagittal section and (**b**) transverse section. Ultrasound imaging detects a clear boundary, solid and hypoechoic mass which located at the carotid bifurcation. (**c**) CDU images of the CBT with a richly vascular and (**d**) with inner low-resistance arterial spectrum. (**e**) Multi slice spiral CTA with 3D VR shows a solid high vascular mass, causing widening of the carotid bifurcation and infiltrating internal carotid artery. (**f**) MRA images shows the typical separation of the external and the internal carotid arteries of the CBT, with heterogeneous enhancement

On ultrasonography, 61 of the 67 (91.0%) lesions were mainly hypoechoic and six (9.0%) were moderately echoic or hypoechoic (Table 3); most lesions had homogeneous echoes (55/67, 82.1%) and clear boundaries (59/67, 88.1%) (Fig. 1a,b). The lesions were of different sizes, with maximum diameters of approximately 1.30 to 7.00 cm. In group B, the maximum diameter, tumor volume, horizontal distance from the upper margin to the mastoid, and distance from the upper margin to the mandibular angle were all larger than those in group A, indicating that Shamblin type III CBTs were larger than types I and II and closer to the base of the skull. When comparing groups A and B, there was no significant difference in blood flow. Rich, moderate, and small blood signals were observed for 34 (50.8%), 22 (32.8%), and 11 (16.4%) of the 67 lesions (Fig. 1c). Pulsed wave doppler (PWD) detected a low-resistance blood flow spectrum (Fig. 1d). CTA showed whether lesions had clear uniform or heterogeneous enhancement (Fig. 1e). MRA showed that the lesions had mixed T1 and slightly longer T2 signals and uniform or heterogeneous enhancement (Fig. 1f).

**Table 3:** Comparison between the two groups of the radiographic parameters of the carotid body tumors

		Group A (n = 11)	Group B (n = 56)	*Z*/*χ*^2^	*P*
Maximum diameter (cm)		2.30 (2.10, 2.90)	4.10 (3.20, 5.33)	4.395	<0.001
Volume (cm^3^)		4.90 (4.40, 8.97)	28.22 (14.38, 40.45)	−4.114	<0.001
Horizontal distance from the upper margin to the papillae (cm)		−3.31 (− 3.95,−1.74)	−1.71 (− 2.54,−1.22)	−2.827	0.005
Horizontal distance from the upper margin to the mandibular angle (cm)		0.55 (− 0.41, 1.93)	1.79 (1.00, 2.50)	−2.573	0.010
Fully surrounded	Yes	0 (0.0)	49 (87.5)		0.000
	No	11 (100.0)	7 (12.5)		
Fuzzy boundary	Yes	1 (90.9)	7 (12.5)		1.000
	No	10 (9.1)	49 (87.5)		
Low-level echo	Yes	10 (90.9)	51 (91.1)		1.000
	Deny	1 (9.1)	5 (8.9)		
Ultrasound blood flow grade	Level 1	2 (18.2)	9 (16.1)	0.367	0.911
	Level 2	4 (36.4)	18 (32.1)		
	Level 3	5 (45.4)	29 (51.8)		

### Histopathology

Macroscopically, the CBTs mainly presented as dark-red solid masses surrounding the common carotid artery bifurcation and internal and external carotid arteries. The tumors had no capsules and closely adhered to the artery wall. The blood supply was extremely rich, and abnormal vasculature could be observed on the surface.

**Fig. 2:**
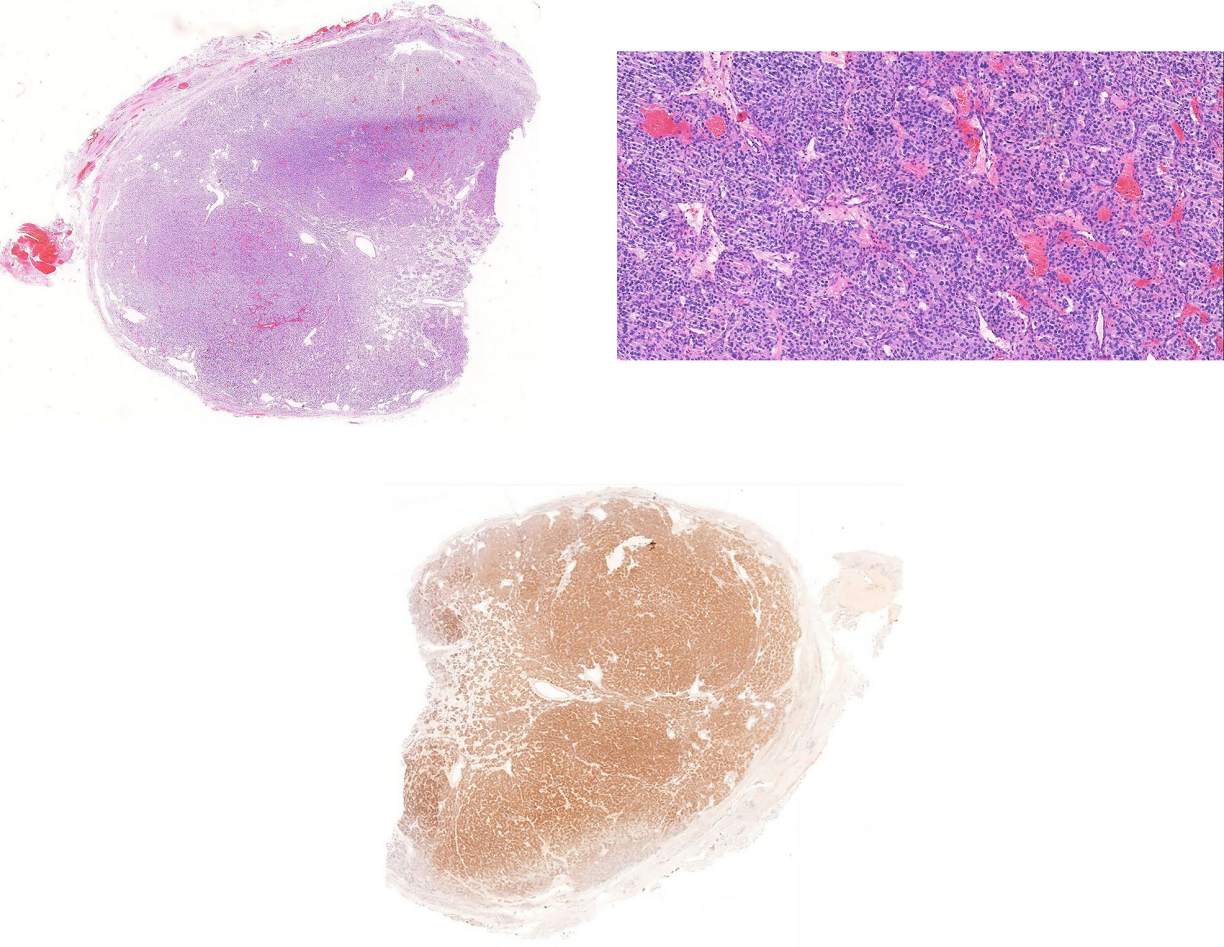
Pathological and immunohistochemical images. (**a**, **b**) Pathological section of CBT, (**b**) HE20x, boundary is relatively clear, the cell nest size is consistent, and there is no significant increase. The tumor cells have moderate atypia, some of which have strange nuclear characteristics, and nuclear division is not easy to see. (**c**) Immunohistochemical sections of CBT (Ret)

Sixty-seven cases of CBT paraganglioma were diagnosed. Most tumors had clear boundaries, no clear capsule or vascular invasion, similar-sized tumor cell nests, no significant enlargement, no significant decrease in the number of supporting cells, scarce stroma, rich tumor cell periplasm, occasional appearance of pathological nuclear division, no tumor necrosis, and no obvious manifestations of stiffening (Fig. 2a,b).

Immunohistochemical examinations were performed in 61 cases, and the main phenotypes included Ki-67, SDHB, SSTR 2, SSTR 5, Syn, CgA, CD34, SMMHC, NF, Ret, P53, GATA-3, and INSM 1. Sertoli cells were positive for S-100 (Fig. 2c). Molecular pathological examination was performed in five cases: missense mutations were found in one BRAF exon 15 and no mutations in exons 10–11,13–16 of the RET gene. Thirty cases of special staining were positive, mostly for elastic fibers (encasing myogenic vessels), mesh fibers (outlining cell nests), Masson’s trichrome (stroma), and Sirius Red (interstitial).

### Correlation analysis

Table 4 shows the assessment of the correlation between the imaging variables and surgical results. All imaging parameters were correlated with surgical blood loss, blood transfused during surgery, duration of surgery, intraoperative classification and skull-base invasion; preoperative imaging classification was highly correlated with intraoperative classification. With an increase in the size of the CBT, the amount of blood transfused, duration of surgery, intraoperative classification level, and possibility of skull-base invasion increased.

**Table 4:** Correlation analysis of imaging characteristics of carotid body tumors and surgical results [*r* (*P*)]

	Maximum diameter	Volume	Horizontal distance from the upper margin to the papillae (cm)	Horizontal distance from the upper margin to the mandibular angle (cm)	Preoperative imaging classification
Surgical blood loss	0.678 (< 0.001)	0.621 (< 0.001)	0.601 (< 0.001)	0.495 (< 0.001)	0.558 (< 0.001)
Surgical blood transfusion volume	0.422 (< 0.001)	0.428 (< 0.001)	0.360 (0.003)	0.265 (0.030)	0.381 (0.001)
Duration of surgery	0.663 (< 0.001)	0.583 (< 0.001)	0.480 (< 0.001)	0.436 (< 0.001)	0.421 (< 0.001)
Intraoperative classification	0.543 (< 0.001)	0.515 (< 0.001)	0.358 (0.003)	0.334 (0.006)	0.829 (< 0.001)
Skull-base invasion	0.564 (< 0.001)	0.491 (< 0.001)	0.287 (0.018)	0.408 (0.001)	0.407 (0.001)

### Shamblin type III CBT diagnostic threshold

**Fig. 3:**
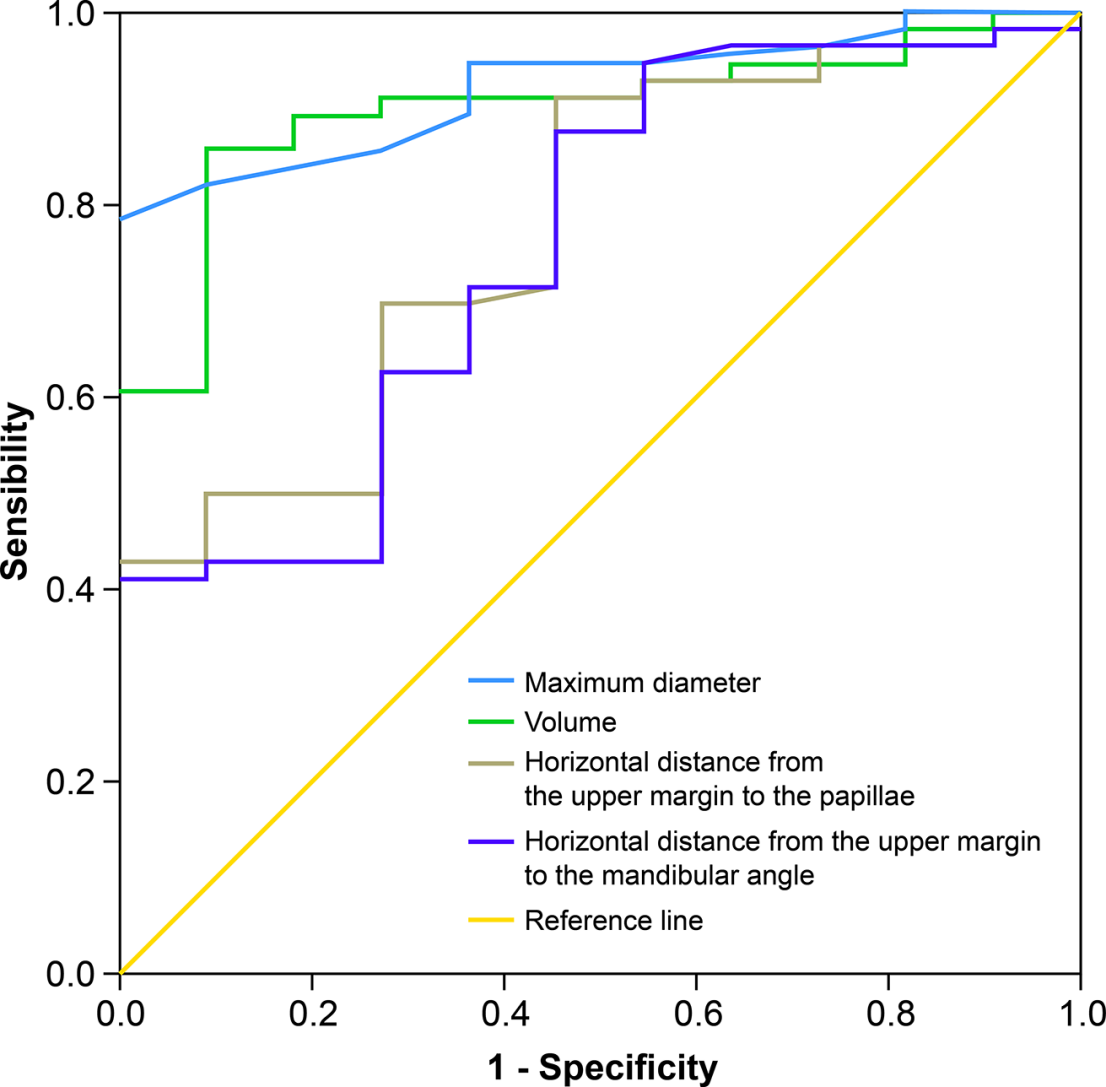
The receiver operating characteristic curve for a Shamblin III carotid body tumor

Using surgical classification as the gold standard, the ROC curves of the maximum diameter of the lesion, tumor volume of the tumor body, distance from the upper margin to the mastoid level, and distance from the upper margin to the mandibular angle yielded areas under the curve values of 0.921, 0.899, 0.775, and 0.756, respectively (Fig. 3; Table 5). All showed good diagnostic results. By selecting several diagnostic parameters for analysis, the maximum diameter of the lesion, tumor volume, horizontal distance from the upper margin to the mastoid, and distance between the upper margin to the mandibular angle of the tumor were 3.10 cm, 10.15 cm^3^, − 3.26 cm, and 0.57 cm, respectively. When the diagnostic efficacy was high, the Youden index was the greatest, indicating the best diagnostic threshold.

**Table 5:** Carotid body tumor diagnosis thresholds for Shamblin type III

Measurement index	Diagnostic boundaries	Sensitivity (%)	Specificity (%)	Youden index (%)
Maximum diameter	3.10 cm	0.821	1.000	0.786
Volume	10.15 cm^3^	0.857	0.909	0.766
Horizontal distance from the upper margin to the papillae	−3.26 cm	0.911	0.545	0.456
Horizontal distance from the upper margin to the mandibular angle	0.57 cm	0.875	0.545	0.420

## Discussion

Sporadic CBTs are mostly unilateral lesions, while familial ones are mostly bilateral [4]. Patients with a CBT are usually 30 to 60 years old and exhibit unilateral onset; the incidence of this tumor is < 20% [1]. In this study, the patients ranged in age from 15 to 77 years, with a mean age of 43.00 ± 13.34 years, and nine patients (approximately 13.64%) had bilateral onset.

CBT pathogenesis is poorly understood and may be associated with chronic hypoxic stimulation of the carotid body. Familial incidence may be related to mutations in the succinate dehydrogenase gene [1] as succinate dehydrogenase deficiency makes patients prone to hypoxia, resulting in carotid body hyperplasia and tumor formation. In this study, the incidence of CBT was similar in males and females, and no obvious difference between Shamblin type and sex was found, which is consistent with previous studies [1, 5]. However, more female than male patients are found in high-altitude areas, which may be associated with long-term low oxygen environments [1, 5].

CBTs are low-incidence, slow-growth, benign tumors, but have malignant tendencies. The incidence of malignant CBTs is approximately 2–9% and, typical of the biological behavior observed histologically between malignant morphology and distant metastasis, approximately 7.5% of CBTs occur with cervical lymph node metastasis [6]. Pathologically, all 66 patients in this study had benign tumors and no local lymph node metastases were identified. Patients with CBTs may be asymptomatic for a long time, and tumor enlargement that compresses adjacent blood vessels and nerves can have clinical manifestations. Approximately one in ten patients with CBTs can develop peripheral vascular and nerve invasion of the tumor, which can cause dysphagia, hoarseness, and Horner syndrome symptoms, and carotid sinus allergic reactions in patients with slow heart rate, hypotension, limited cardiac function, cerebral ischemia–hypoxia, and syncope [7]. The main clinical manifestations observed in this study included pharyngeal discomfort, cough, choking cough, swelling of the neck, dizziness, headache, and dyspnea; however, no significant differences were found in the clinical symptoms of the patients.

Imaging examinations of CBTs include CDU, CTA, MRA, and digital subtraction angiography. CTA can help understand the tumor and its surrounding tissues through three-dimensional imaging. MRA tissue resolution is high and can be used to observe fluid spatial effects. CTA and MRA, as common examination methods for preoperative diagnosis, can provide accurate vascular imaging information and are used to evaluate tumor size, morphology, and relationship with the carotid artery and surrounding tissues [8, 9]; however, the cost is high and they are contraindicated in some circumstances. Digital subtraction angiography is the gold standard for CBT diagnosis, as it enables the tumor’s blood supply to be observed and preoperative vascular embolization to be performed. Its disadvantages include invasiveness and high risks of allergy, bleeding, and embolism. CDU is simple, safe, noninvasive, and reproducible, and with ultrasound imaging technology developing rapidly, it is the preferred method to screen for, diagnose, and follow up CBTs. Using CDU, tumor size, location, blood flow, and peripheral tissue can clearly be observed through multi-angle surface scanning, thus providing reliable information for clinical diagnosis. This study’s results showed that CBTs were usually located at the bifurcation of the common carotid artery, mostly as a round or oval mass partially or completely surrounding the bifurcation of the common carotid artery and the internal and external carotid arteries. Their presence causes an increased angle between the internal and external carotid arteries and pressure displacement of the artery.

Ultrasonography mainly shows CBTs as homogeneous hypoechoic masses with clear boundaries, rich blood flow signals in the periphery and interior of the mass, a Doppler spectrum, high speed, and low resistance. The lesions show uniform or heterogeneous enhancement on CTA imaging. MRA showed that the lesions had slightly longer T2 signals, and the enhancement examination showed uniform or uneven enhancement. CBTs are mainly supplied with blood from the external carotid artery, and the blood supply is extremely rich; malformed vascular networks can be observed on the surface, with many micro-arteriovenous fistulas and very rapid blood circulation [4]. Gao et al. [2] used contrast-enhanced ultrasound (CEUS) to observe enhancement patterns of CBTs and evaluate tumor infiltration of the carotid artery wall. Their results showed that CBTs in the arterial and venous stages display uneven high enhancement, a delayed period of enhancement, no carotid artery wall with no obvious enhancement, and a fuzzy boundary to the carotid artery wall with uneven high enhancement performance. The preoperative evaluation of CBTs is a novel idea; however, the number of patients is small and the diagnostic value unclear [2].

CBT ultrasonography findings show mostly solid hypoechoic masses that require differential diagnosis from other solid lesions that may occur at this site, such as neurogenic tumors and lymphadenopathies. Lymphadenopathy is usually characterized by multiple enlarged lymph nodes, uniform and mild enlargement, normal morphology, and visible portal-like structures. Patients with lymph node tuberculosis may present with low fever symptoms, disappearance of the portal structure, internal calcification, liquefaction, and edema of the surrounding tissues. Malignant lymphoma and lymph node metastases are characterized by painless lymph node enlargement, abnormal morphology, and disappearance of the portal structure. Ultrasound manifestations of malignant lymphoma include hypoechogenicity, enhanced posterior echo, grid-like hyperechogenicity, and rich blood-flow signals. The appearance of head and neck lymph node metastases is closely related to the primary lesions. Most primary lesions are nasopharyngeal and thyroid carcinomas, in which the distribution of blood flow signals is disorderly, increased, and thickened, and the blood flow resistance index increased. Neurofibromas and schwannomas are common neurogenic tumors. Single or multiple neurofibromas originating from nerve fibers exhibit central growth. Schwannomas originate from Schwann cells of the nerve sheath membrane, which show eccentric growth and multiple single hairs. Both show solid hypoechogenicity, and the two ends are usually connected to the nerve. When the solid lesion is large, its relationship with the nerve is difficult to show; however, the carotid artery is mostly displaced, whereas the angle between the internal and external carotid arteries does not change significantly. When performing differential diagnosis, attention should be paid to the contact of the mass and the change in angle between the internal and external carotid arteries [10, 11].

The Shamblin classification is widely used for CBTs. This system can, to some extent, predict intraoperative vascular injury risk and postoperative complications, and thus be used in clinical practice to guide surgical strategy [3, 12]. However, Shamblin classification does not fully consider infiltration of the carotid artery wall, including some type I CBT cases. Luna-Ortiz et al. further classified Shamblin type III into classes IIIa and IIIb: tumors classed as IIIb include CBTs that infiltrate the carotid artery wall but have a small body. This classification is poorly associated with perioperative complications and has limited clinical application [13]. The Shamblin classification also does not consider vertical growth in predicting perioperative complications and postoperative cranial nerve damage. Vertical growth of tumors may affect intraoperative treatment of the cranial nerve; for every 1-cm decrease in the distance between CBT and the skull base, the risk of postoperative neurological complications increases by 1.5 times [12, 14, 15]. Using Shamblin classification, the evaluation of tumor contact with the carotid wall and vertical growth includes the following: type I, the upper margin of the tumor is below the mandibular angle; type II, type III, and type IV, the upper margin of the tumor is above the mandibular angle, but below the mastoid tip, and the internal and external carotid arteries; and type V, the upper margin is above the mastoid tip. No wrapping, partial wrapping, and complete wrapping are defined as < 50%, 50%–<100%, and 100%, respectively. This modified classification correlates with the surgical outcomes of CBT and facilitates the choice of clinical surgical options [16].

CBT treatment options include surgical resection, and preoperative embolization and radiotherapy [1]. Preoperative embolization helps reduce the complications associated with bleeding and resection of giant tumors, but is complicated and costly, and may lead to inflammation and carotid artery injury, increasing the risk of ischemic stroke. No consensus has been reached regarding indications for preoperative embolization [17]. Radiotherapy is often used as an adjuvant therapy for inoperable patients, while surgical resection is currently the recommended clinical treatment option. The difficulty of tumor resection increases with increased tumor size, local infiltration, and carotid artery contact; therefore, we advocate early surgical resection to reduce invasion of the peripheral blood vessels and nerves. The possibility [16] of a carotid shunt and artificial vascular internal carotid artery reconstruction should be considered before large CBT excision. Complications may arise, such as bleeding, vascular injury, pseudoaneurysm, and nerve injury [18]. When the tumor diameter exceeds 5 cm, the surgical complication rate can exceed 60%. This study showed a high correlation between pre- and intraoperative imaging classifications. Compared to the maximum diameter, the average tumor diameter has many influencing factors and poor reproducibility, making it unsuitable as a diameter evaluation index. With increases in lesion size, amount of blood transfused, and operation time, increases are observed in the intraoperative classification level and possibility of skull-base invasion, and the number of lesions invading the skull base in Shamblin type III CBTs increases by approximately 66.1%. This is consistent with the results of most previous studies [2, 16].

This study had some limitations. This was a single-center study in which only patients who underwent CDU, CTA, and MRA were included, possibly leading to selection bias. The number of patients with Shamblin type I CBTs was small. This study was retrospective, and CEUS features of the lesions were not included in the analysis; therefore, a prospective study with more patients should be conducted to evaluate the utility of using combined imaging technology in the diagnosis and treatment of CBTs.

## Conclusion

CDU combined with CTA and MRA can accurately assess the size, vertical growth, and degree of contact with the carotid artery of CBTs; provide reliable imaging information for clinical use; and contribute to the selection and determination of surgical strategies. This method has important value in clinical applications.

## Data Availability

The datasets used and/or analysed during the current study are available from the corresponding author on reasonable request. All of the material is owned by the authors and no permissions are required.

## Abbreviations

CBT: carotid body tumor
CDU: color Doppler ultrasonography
CEUS: contrast-enhanced ultrasound
CTA: computed tomography angiography
MRA: magnetic resonance angiography
ROC: receiver operating characteristic
